# Self-Assessment Scales for Depression Screening: A Review of Recent Trends

**DOI:** 10.1192/j.eurpsy.2025.679

**Published:** 2025-08-26

**Authors:** A. Argyriadis, E. Kotrotsiou, S. Kotrotsiou, D. Katsarou, A. Patelarou, E. Patelarou, M. Efstratopoulou, A. Argyriadi

**Affiliations:** 1 Nursing, Frederick University, Nicosia, Cyprus; 2 Nursing, University of Patras, Patra; 3 Department of Preschool Education Sciences and Educational Design, University of the Aegean, Rhodes; 4 Nursing, Hellenic Mediterranean University, Heraklion, Greece; 5 United Arab Emirates University, Al Ain, United Arab Emirates

## Abstract

**Introduction:**

Depression significantly impacts quality of life, and the growing global mental health burden necessitates effective strategies for early detection. Traditional diagnostic methods often involve clinician-led interviews and assessments, which can be time-consuming and may not always be accessible to individuals in underserved or remote areas. As a result, self-assessment scales have emerged as a valuable tool for initial depression screening, offering a cost-effective and timely alternative that empowers individuals to monitor their mental health independently.

Recent trends in self-assessment tools for depression highlight the development of digital platforms, such as smartphone apps and web-based applications, which allow for greater reach and real-time data collection.

**Objectives:**

The primary objective of this study is to systematically review recent trends in the development and use of self-assessment scales for depression screening. With the growing global prevalence of depression and the necessity for early detection, self-assessment tools have become a widely adopted method for screening, offering the advantage of accessibility, cost-effectiveness, and user autonomy. This study aims to evaluate these tools in terms of their psychometric properties, including reliability, validity, and sensitivity, which are crucial for ensuring accurate and dependable depression detection.

**Methods:**

A systematic review methodology was employed, focusing on studies published between 2015 and 2024. The sample included 40 peer-reviewed articles sourced from academic databases, with studies chosen based on their relevance to self-assessment depression screening tools. The sampling strategy involved selecting scales used in diverse settings, including clinical environments, schools, and online platforms. Key tools such as the Patient Health Questionnaire (PHQ-9), Beck Depression Inventory (BDI), and the Depression Anxiety Stress Scales (DASS-21) were analyzed.

**Results:**

Results indicated an increase in the utilization of digital and app-based self-assessment tools, with advancements in adaptive testing and machine learning-based algorithms improving the accuracy and sensitivity of depression screening. Additionally, the results showed promising psychometric reliability and validity across different cultural contexts. However, the study also highlights challenges, including the potential for over-reliance on self-reporting and the underrepresentation of marginalized populations in the development of these tools.

**Conclusions:**

Despite the significant advancements in self-assessment scales for depression screening, challenges remain in ensuring that these tools are both equitable and inclusive. One critical area for future research involves addressing cultural and demographic biases that may limit the effectiveness of these scales in diverse populations.

**Disclosure of Interest:**

None Declared

